# Transgenic Sugarcane Resistant to *Sorghum mosaic virus* Based on Coat Protein Gene Silencing by RNA Interference

**DOI:** 10.1155/2015/861907

**Published:** 2015-01-22

**Authors:** Jinlong Guo, Shiwu Gao, Qinliang Lin, Hengbo Wang, Youxiong Que, Liping Xu

**Affiliations:** ^1^Key Laboratory of Sugarcane Biology and Genetic Breeding, Ministry of Agriculture, Fujian Agriculture and Forestry University, Fuzhou 350002, China; ^2^National Research and Development Center for Sugarcane Industry Technology, Fujian Agriculture and Forestry University, Fuzhou 350002, China

## Abstract

As one of the critical diseases of sugarcane, sugarcane mosaic disease can lead to serious decline in stalk yield and sucrose content. It is mainly caused by *Potyvirus sugarcane mosaic virus* (SCMV) and/or *Sorghum mosaic virus* (SrMV), with additional differences in viral strains. RNA interference (RNAi) is a novel strategy for producing viral resistant plants. In this study, based on multiple sequence alignment conducted on genomic sequences of different strains and isolates of SrMV, the conserved region of coat protein (CP) genes was selected as the target gene and the interference sequence with size of 423 bp in length was obtained through PCR amplification. The RNAi vector pGII00-HACP with an expression cassette containing both hairpin interference sequence and *cp4-epsps* herbicide-tolerant gene was transferred to sugarcane cultivar ROC22 via *Agrobacterium*-mediated transformation. After herbicide screening, PCR molecular identification, and artificial inoculation challenge, anti-SrMV positive transgenic lines were successfully obtained. SrMV resistance rate of the transgenic lines with the interference sequence was 87.5% based on SrMV challenge by artificial inoculation. The genetically modified SrMV-resistant lines of cultivar ROC22 provide resistant germplasm for breeding lines and can also serve as resistant lines having the same genetic background for study of resistance mechanisms.

## 1. Introduction

Sugarcane (*Saccharum* spp. L.), a major sucrose accumulator and biomass producer, is one of the most important field crops grown in the tropics and subtropics [1]. It accounts for 92% of all sugar produced in China [2] and 80% of that in the world. Sugarcane mosaic disease is one of the most serious sugarcane diseases. It primarily damages chloroplasts, blocks photosynthesis, and decreases photosynthetic products, thus resulting in a decline in yield and sugar content [2]. Sugarcane mosaic disease is caused by the sugarcane mosaic virus subgroup of* Potyvirus sugarcane mosaic virus* (SCMV) and/or* Sorghum mosaic virus* (SrMV) [3].* Potyvirus* is a single-stranded RNA virus, with simple genome structure encoding 10 mature proteins, named from N-terminal to C-terminal: the first protein (P1), helper component proteinase (HC-pro), the third protein (P3), the first 6K protein (6K1), cylindrical inclusion protein (CI), the second 6K protein (6K2), viral protein genome-linked (VPg), nuclear inclusion a protein (NIa), nuclear inclusion b protein (NIb), and coat protein (CP) [4].

The virus strain differentiation is complex, with both of the virus members that cause sugarcane mosaic disease having several different virus strains [5]. At least eight stains have been reported [6], including five from SCMV and three from SrMV. The mixed infection of different virus strains also occurs [3, 6, 7], and dominant virus strains are variable [8]. In the 1980s there were at least three strains of SCMV including strains A, D, and E in mainland China [9]. However, the dominant pathogen has become strain H of SrMV in the last ten years [10]. The simplicity of pathogenic virus genome quickens the change of dominant strains. Coupled with the complexity of the genetic background of sugarcane, the difficulty in the crossbreeding of virus-resistant varieties is obvious, especially for breeding sugarcane varieties resistant to multiple virus strains.

Improving plant antiviral resistance by gene silencing has proven to be effective in several plant-virus biosystems. Abel et al. first transferred CP genes of* tobacco mosaic virus* (TMV) into tobacco and successfully obtained anti-TMV tobacco plants [11]. Subsequently, different genes in* Potyvirus* genome were introduced into various plants to obtain corresponding resistant plants [12]. Joyce et al. introduced the CP gene of SCMV into sugarcane, and the CP-transformed plants displayed various phenotypes after SCMV challenge [13]. Ingelbrecht et al. introduced the CP gene of SrMV-H strain into sugarcane and obtained a range of different resistance types [14]. Yao et al. transferred the CP gene of SCMV-E strains into* S. officinarum* Badila and obtained SCMV-resistant transgenic lines but, after field experiments, found that some of them showed symptoms of mosaic disease, which were shown to be infected with SrMV-H and SCMV by RT-PCR [15]. Therefore, resistance performance of transgenic offspring obtained by the introduction of complete CP genes is complex, and resistance loss to the same or different virus strains in transgenic plants suggests that an improved method is necessary.

Gene silencing through RNA interference (RNAi) appears to be present in most eukaryotic organisms. Homologous RNA is degraded with the introduction of double-stranded RNA (dsRNA), which can lead to target gene silencing [16]. The target gene to be silenced can include a single gene or part sequence of a single gene that is targeted for suppression or can include multiple consecutive segments of a target gene, multiple nonconsecutive segments of a target gene, multiple alleles of a target gene, or multiple target genes from one or more species. RNAi-based antiviral breeding appears to be a promising strategy for development of virus resistance transgenic plants. There have been many successful examples of RNAi-mediated virus resistance improvement in crops, such as soybean [17], tobacco [18, 19], potato [20], barley [21], tomato [22], maize [23], and rice [24]. In sugarcane, the application of RNAi technology to suppress lignin biosynthesis was reported [25, 26], but no study on the application of RNAi technology in improving disease resistance has been reported. SrMV, the pathogen of sugarcane mosaic disease, is a single-stranded RNA virus, which replicates using a viral RNA polymerase. Viral genes in the form of dsRNA generate during replication, which is the basis of using RNAi technology for its control.

In this study, we have used RNAi technology, taking highly conserved sequences of SrMV CP gene as a silencing target, and RNAi expression vector with hairpin structures and introduced them into sugarcane via* Agrobacterium*-mediated transformation. We then performed screening and biological identification to obtain anti-*Sorghum mosaic virus* transgenic sugarcane plants. This study provides sugarcane transgenic lines with different resistances in the same genetic background for study of resistance mechanisms and for breeding of multiresistance to various SrMV strains.

## 2. Materials and Methods

### 2.1. Bacterial Strains and Plasmids

*Escherichia coli* strain DH5A,* Agrobacterium tumefaciens* strain EHA105, and intermediate vector pHANNIBAL were provided by the Key Laboratory of Sugarcane Biology and Genetic Breeding, Ministry of Agriculture (Fuzhou, China). The glyphosate tolerance gene* cp4-epsps* was obtained from roundup ready soybean by PCR and verified by sequencing, and the intermediate vector pGIIHA containing 35S promoter-*cp4*-*epsps*-CaMV polyA cassette was constructed subsequently in previous study.

### 2.2. Reagents and Plant Materials

Reverse transcriptase (AMV), restriction endonucleases, T4 DNA ligase, and PCR kits were purchased from Fermentas (USA); dephosphorylation (BAP) kit was purchased from Takara (Dalian, China); Wizard DNA clean-up kit gel extraction kit was purchased from Promega Corporation (USA); plant genomic DNA extraction kit was purchased from TIANGEN (Beijing, China); components in MS medium were purchased from Sangon (Shanghai, China); Trizol reagents were purchased from Invitrogen (USA); Timentin disodium salt and 2,4-dichlorophenoxyacetic acid (2-4-D) were purchased from Sigma (USA); and herbicide (47% isopropylamine salt of N-glycine) applicable by foliar spraying was purchased from Sannong Co., Ltd. (Fujian, China). ROC22 was the most popular cultivar in China, which was provided by the Key Laboratory of Sugarcane Biology and Genetic Breeding, Ministry of Agriculture (Fuzhou, China).

### 2.3. Medium

Infection medium M1 is 1/2 MS + 100 *μ*mol/L acetosyringone + 20 g/L sucrose, pH 5.8; cocultivation medium M2 is 1/2 MS + 3.0 mg/L 2.4-D + 100 *μ*mol/L acetosyringone + 20 g/L sucrose + 5 g/L agar powder, pH 5.8; subculture medium M3 is MS + 3.0 mg/L 2.4-D + 300 mg/L Timentin + 8.0 mg/L herbicide + 30 g/L sucrose + 6 g/L agar powder, pH 5.8; differential medium M4 is MS + 2.0 mg/L BA +0.5 mg/L KT + 0.2 mg/L NAA + 300 mg/L Timentin + 6.0 mg/L herbicide + 30 g/L sucrose + 6 g/L agar powder, pH 5.8; rooting medium M5 is 1/2 MS + 0.2 mg/L 6-BA + 3 mg/L NAA + 60 g/L sucrose + 6 g/L agar powder, pH 5.8.

### 2.4. RNAi Target Sequence Selection

Genome sequences of the SrMV strains (H, I, and M) isolated from sugarcane were collected from GenBank. Using DNAMAN 5.22 software (http://www.lynnon.com/), multiple sequence alignment was performed to determine the most conservative nucleic acid segment as RNAi target sequence. The fast alignment was generated using DNAMAN 5.22 with default parameters (Gap penalty was set at 7, K-tuple at 3, and number of Top at 5). The accession numbers of the chosen sequences in alignment were EU189035, EU189036, EU189037, EU189041, EU189042, EU189038, EU189043, EU189044, EU189045, EU189046, EU189039, EU189040, U07219, AJ310198, NC004035, and SMU57358 and SrMV FZ strain was kept in our lab (a SrMV strain isolated from Fuzhou, China, unsubmitted).

### 2.5. Interference Fragment Preparation and Hairpin Intermediate Vector Construction

According to multiple sequence alignment results, a pair of specific primers targeting the most conservative segment were designed, with extra* Xba* I and* Xho* I endonuclease restriction sites on the 5′ end of the forward primer CPS and* Cla* I and* Kpn* I on the 5′ end of the forward primer CPA. The primer sequences are as follows: 

CPS:

5′-GA$\underset{Xba\;\;\text{I}}{\underline{\text{TCTAGA}}}\;\underset{Xho\;\;\text{I}}{\underline{\text{CTCGAG}}}$TGTTTGGACAATGATG-3′

CPA:

5′-CT$\underset{Cla\;\;\text{I}}{\underline{\text{ATCGAT}}}\;\underset{Kpn\;\;\text{I}}{\underline{\text{GGTACC}}}$GCACATCAGTGGTTCT-3′

Target sequence for RNAi was amplified by PCR using SrMV FZ as template. The 50 *μ*L PCR reaction mix contained 5.0 *μ*L 10 × PCR buffer, 4.0 *μ*L deoxynucleotide triphosphates (dNTPs) (2.5 mM), 2.0 *μ*L each of forward and reverse primers (10 *μ*M), 2.0 *μ*L template (100 ng), and 0.25 *μ*L Ex-Taq enzyme (5 U/*μ*L). The ddH_2_O was added as supplement. The PCR amplification program consisted of predenaturation for 5 min at 94°C, denaturation for 30 s at 94°C, annealing for 30 s at 60°C, and extension for 30 s at 72°C for 30 cycles; final extension was for 10 min at 72°C. The PCR product was purified by gel extraction kit to prepare for digestion. The PCR product was separated in 2% agarose gel. The target DNA fragments were excised and purified using an agarose gel purification kit. Using the two sets of restriction enzymes,* Cla* I/*Xba* I and* Xho* I/*Kpn* I, successively, the target sequence was inserted into the two sides of the intronic region of pHANNIABL vector. A clone with a recombinant plasmid was validated by PCR, double digestion, and sequencing and was termed as pHANNIABL-CP.

### 2.6. Construction of the RNAi Expression Vector

The pGIIHA intermediate vector was digested with* Not* I and then purified by gel extraction kit. The purified products were dephosphorylated according to manual of the dephosphorylation (BAP) kit.* Not* I-digested hairpin interference cassette fragments from pHANNIBAL-CP were inserted into the* Not* I site of pGIIHA. A clone with a recombinant plasmid was validated by PCR, double digestion, and sequencing and was termed as pGII00-HACP.

### 2.7. Preparation of the Engineering Bacteria

According to the freeze-thaw method reported by Holsters et al. [27], the RNAi vector pGII00-HACP was transformed into* A. tumefaciens* EHA105. The positive clone identified by PCR was inoculated into the LB medium containing kanamycin (50 *μ*g·mL^−1^) and rifampicin (35  *μ*g·mL^−1^) for shake culture at 150 rpm at 37°C. When OD_600_ reached 1.0 to 1.2, the culture was centrifuged at 5,000 rpm for 5 min at room temperature to discard the supernatant. The pellet was collected and resuspended with M1 medium and then centrifuged at 5,000 rpm for 5 min at room temperature again to discard the supernatant. The pellet thus obtained was resuspended and diluted with M1 medium to OD_600_ = 1.0.

### 2.8. *Agrobacterium*-Mediated Transformation and Screening

Leaf explants from sugarcane ROC22 were cultured on MS medium supplemented with 3.0 mg/L 2,4-D for one week and then cocultivated with recombinant* A. tumefaciens* EHA105 for 30 min. The plant tissue was picked out and sucked dry with filter paper and cultured on M2 medium for 2-3 d; then the plant tissue was transferred to M3 culture medium and screened for 2-3 generations. After that, the plant tissue was transferred to M4 differential medium, followed by a period of culture on M5 rooting medium when the tissue culture seedlings grew to 4~5 cm in length. The seedlings were then transferred to 72-well nutrition plate when their roots reached about 2.5 cm. After the seedlings were transplanted, the plants were sprayed with liquid herbicide solutions at the concentrations of 3.0‰ and survivors from the herbicide treatments were selected.

### 2.9. PCR for Positive Identification

Genome DNA extracted from herbicide-resistant and control plants was diluted to a concentration of 50 ng/*μ*L and was used as PCR template. 35S promoter and* cp4-epsps* gene were selected as the target genes for identification. Genome DNA of the herbicide-resistant plants was isolated and tested by PCR, using pGII00-HACP as a positive control, genome DNA of untransformed plants as a negative control, and ddH_2_O as a blank control.

The primer sequences for the 35S promoter were 35SPro F: 5′-TCTAACAGAACTCGCCGTGAA-3′ and 35Spro R: 5′-AAGGGTCTTGCGAAGGATAGT-3′, and the primers sequences for the* cp4-epsps* gene were cp4-epsps F: 5′-GTCCTTCATGTTCGGCGGTCTC-3′ and cp4-epsps R: 5′-ACGTCGATGACTTGGCTGGTGA-3′. The 50 *μ*L PCR reaction mix contained 5.0 *μ*L 10 × PCR buffer; 4.0 *μ*L deoxynucleotide triphosphates (dNTPs) (2.5 mM); 2.0 *μ*L each of forward and reverse primers (10 *μ*M); 2.0 *μ*L plasmid DNA (100 ng); and 0.25 *μ*L Ex-Taq enzyme (5 U/*μ*L). The sterile ddH_2_O was added as supplement. The PCR amplification program consisted of predenaturation for 5 min at 94°C, denaturation for 30 s at 94°C, annealing for 30 s at 57°C, and extension for 30 s at 72°C for 30 cycles, and final extension was for 10 min at 72°C. The PCR products were separated by 2.0% agarose gel electrophoresis, and the results were analyzed by gel imaging and analysis system.

### 2.10. Identification of SrMV Resistance by Artificial Inoculation

According to Gómez et al. [28], sugarcane leaves with typical mosaic symptoms were collected and diagnosed with SrMV infection. The artificial inoculation method according to Użarowska et al. [29] used the SrMV-positive leaf samples as the virus infection source.

## 3. Results and Analysis

### 3.1. RNAi Target Sequence Selection

**Figure 1:**
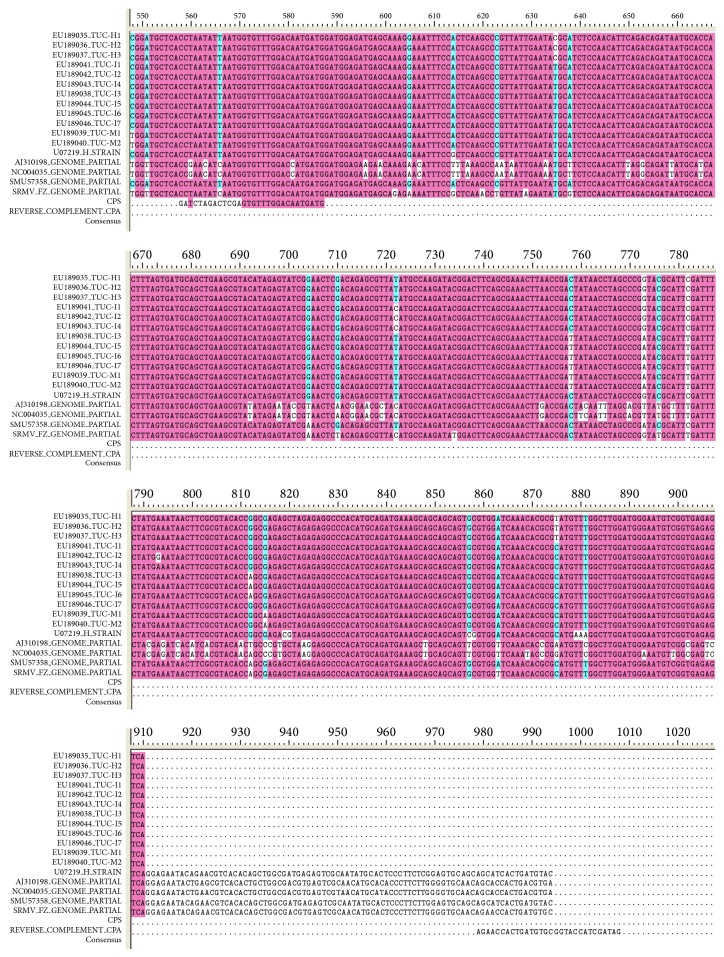
Multiple sequence alignment of SrMV* CP* genes.

Thirteen sequences of the SrMV CP gene and four sequences of the SrMV whole genome in NCBI were selected for multiple sequence alignment analysis.  Figure 1 showed that CP genes with 87.39% homology were the most conservative fragment in SrMV genome sequence. Therefore, a conserved region of 423 bp, from 573 bp to 995 bp in CP genes, was identified as the interference fragment sequence.

### 3.2. Interference Fragment Preparation and Hairpin Intermediate Vector Construction

**Figure 2:**
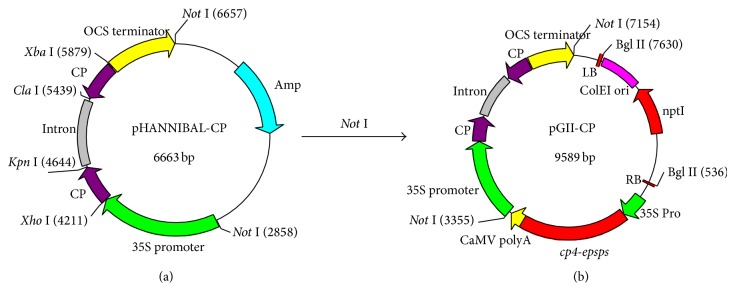
A simplified flowchart showing the construction of binary vectors. (a) Vector diagram of pHANNIBAL-CP; (b) diagram of RNAi expression vector pGII00-HACP.

The target interference fragment with the expected length of 423 bp was obtained by PCR and verified by sequencing. Using the two sets of restriction endonuclease—*Kpn* I/*Xho* I or* Xba* I/*Cla* I, the 423 bp of partial CP gene and its reverse compliment fragment were successively inserted into each intron site contained in pHANNIBAL and enabled the hairpin intermediate vector pHANNIBAL-CP to make a hairpin loop (Figure 2(a)).

### 3.3. Construction of RNAi Expression Vector

Recombinant RNAi expression vector was identified by* Not* I restriction analysis, PCR, and sequencing (data not shown), and the positive hairpin RNAi expression vector was termed as pGII00-HACP (Figure 2(b)).

### 3.4. *Agrobacterium*-Mediated Transformation and Screening

**Figure 3:**
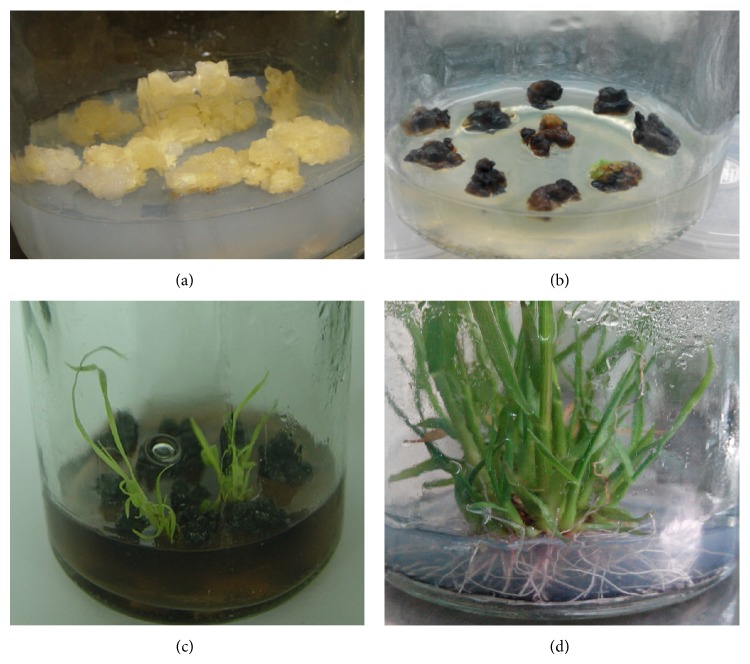
Putative recombinant screening. (a) Wild-type calli, (b) transformed calli screening by herbicide, (c) regenerated seedlings at the stage of differentiation selection culture, and (d) regenerated seedlings at the stage of rooting culture.

The constructed RNAi expression vector pGII00-HACP was transformed into* A. tumefaciens *EHA105 and used to infect sugarcane calli. After coculture, selective subculture and differentiation culture under herbicide stress, and rooting culture (Figure 3), about five hundred regenerated seedlings were obtained.

### 3.5. Herbicide Resistance Screening and PCR Detection of Resistant Regenerated Plants

**Figure 4:**
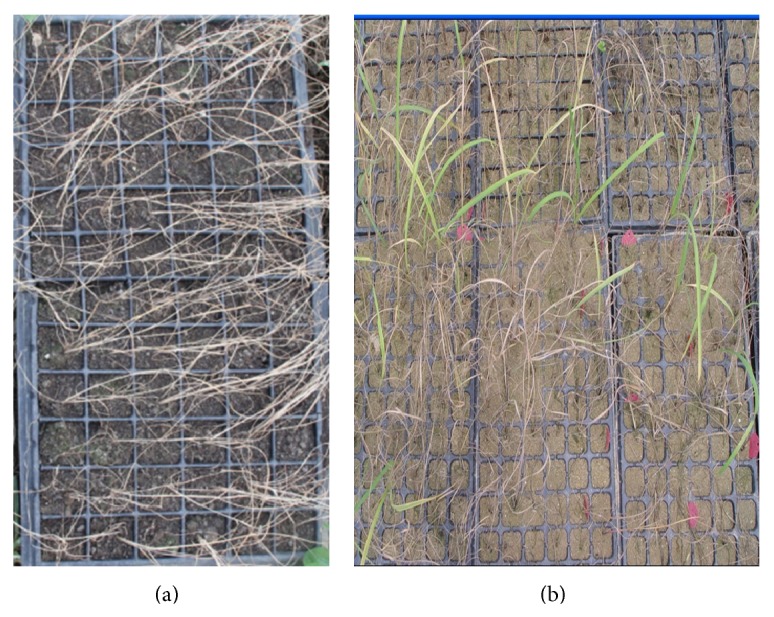
Spraying screening by 0.3% herbicide. (a) Wild-type plants and (b) putative transformants.

**Figure 5:**
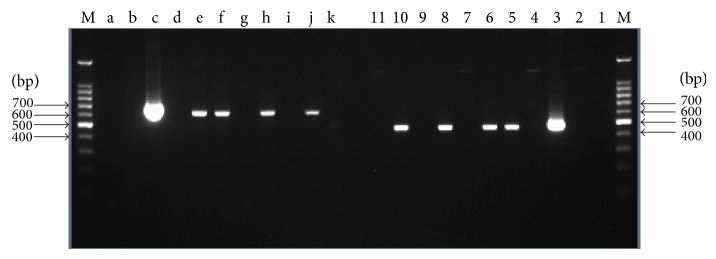
PCR amplification products of* cp4-epsps* gene and 35S promoter. M: DNA marker; d~k: 35S promoter detection; 4~11:* cp4-epsps* gene detection; c and 3: positive control; b and 2: negative control; a and 1: ddH_2_O blank control.

A portion of the regenerated putative recombinants survived herbicide treatment (Figure 4). Among these, 16 plants from 50 survivors were further identified as positive by PCR, exhibiting existence of 463 bp specific band in the 35S promoter detection and 623 bp specific band in the* cp4-epsps* gene detection. In order to get more putative resistant transgenic plants, 0.3% herbicide, which was not a complete lethal concentration for sugarcane, was used in this study, although it led to higher false-positive rate.  Figure 5 showed part of PCR products identified by gel electrophoresis.

### 3.6. Disease Incidence of Artificially Inoculated Transgenic Lines

**Figure 6:**
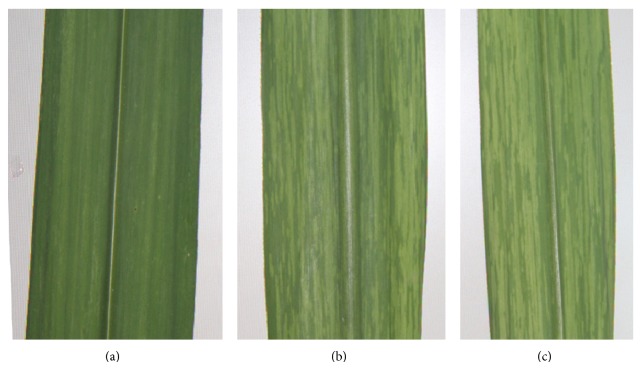
The SrMV-resistant and sensitive symptoms. (a) Transgenic plants had no symptoms; (b) transgenic plants displayed symptoms; (c) nontransgenic control plants displayed appeared symptoms.

**Figure 7:**
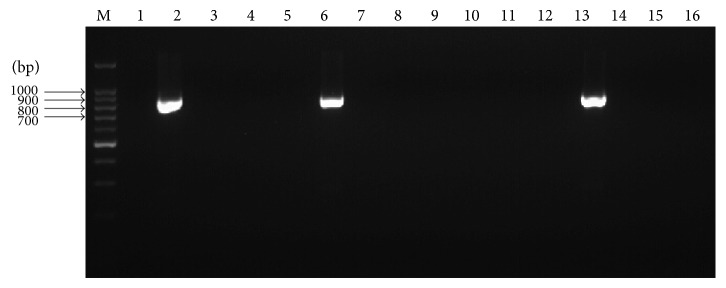
RT-PCR detection of SrMV in experimental plants. M: DNA marker; 1: ddH_2_O blank control; 2: nontransgenic control showed symptoms; 3~6, 8~14, and 16: transgenic plants without symptoms; 7, 15: transgenic plants showed symptoms.

After artificial inoculation with SrMV, 14 transgenic plants showed no symptoms and no virus in RT-PCR detection and were judged to be uninfected after SrMV challenge; two transgenic plants and nontransgenic control plants showed symptoms and SrMV in RT-PCR detection, diagnosed as infected after SrMV challenge (Figures 6 and 7). Therefore, it could be concluded that hairpin RNAi expression vector pGII00-HACP, which resulted in production of resistance against SrMV, was successfully introduced into sugarcane and according to 87.5% transgenic plants showed improved resistance to SrMV.

## 4. Discussion

Mosaic virus-resistant transgenic sugarcane plants have been obtained via particle gun bombardment [14, 15, 30–32], and some transgenic plants were significantly improved in mosaic virus resistance. However, most events produced by gene gun bombardment tend to show high copy numbers of recombinant inserts [33]. Modern sugarcane varieties are a complex allopolyploid and aneuploid genetic background of* S. officinarum* (chromosome number 80) and* S. spontaneum* (chromosome number from 40 to 128), with even* Erianthus arundinaceus* included in sugarcane clones bred during last five years in China [34]. Hence, it was hard to prove clearly characters such as copy numbers, insertion sites, and border sequences in genetically modified (GM) sugarcane via gun bombardment. However, such information is necessary for any GM organisms including GM sugarcane before the application of transgenic field trials. Also high copy numbers of exogenous genes, such as the selective marker, in GM organism can even cause cosuppression [35].

It has been reported that* Agrobacterium*-mediated transformation leads to clean, discrete, low copy, well-defined, unrearranged DNA insertions into the plant genome [36, 37]. However,* Agrobacterium*-mediated transformation is not as successful as gene gun bombardment in sugarcane. In the present study, hairpin RNAi expression vector pGII00-HACP was transferred to sugarcane cultivar ROC22 via* Agrobacterium*-mediated method. The 423 bp interference fragment derived from the most conservative region of the CP gene of SrMV based on multiple alignment analysis of all the three SrMV strains (H, I, and M). The purpose is to obtain multistrains resistant sugarcane plants. In addition,* cp4-epsps *gene contained in pGII00-HACP can be used as a high-efficiency selective marker and also endows sugarcane with a herbicide-tolerant trait. This enables farmers to make the process of weed control more efficient and flexible.

RNAi is a highly conserved dsRNA-guided mechanism that mediates sequence-specific posttranscriptional gene silencing [38]. As a source of dsRNA, plasmid-expressed short hairpin RNA (shRNA) has been demonstrated to be able to trigger RNAi silencing [21–24]. The study of Varsha Wesley et al. [39] showed that intron-containing constructs (ihpRNA) can generally enable 90–100% of independent transgenic plants to show silencing. The average percentages of ihpRNA, hpRNA, cosuppression, and antisense constructs at silencing were 90%, 58%, 13%, and 12%, respectively [39]. pHANNIBAL, an intermediate generic vector used in this study, allows a simple, single PCR product from CP gene to be easily converted into a highly effective ihpRNA silencing construct. Similar to Varsha Wesley et al. [39], the ihpRNA silencing construct targeting SrMV CP gene in this study exhibited 87.5% high silencing effect.

In summary, a 423 bp highly conserved region from the CP gene of SrMV was selected as the interference sequence based on multiple alignment analysis of all the three SrMV strains (H, I, and M) and several other isolates. The hairpin RNAi expression vector pGII00-HACP was transferred to sugarcane cultivar ROC22 via* Agrobacterium*-mediated transformation. After herbicide screening, PCR molecular identification, and artificial inoculation challenge, anti-SrMV positive transgenic lines were successfully obtained. This study provides the foundation for a further study on silencing mechanism of SrMV-CP gene expression based on RNA interference and provides novel materials to evaluate the silencing effect connected with exogenous gene copy numbers and insertion sites. It also provides new material for broad-spectrum antiviral sugarcane breeding.
